# Chronic Unreduced Anterior Shoulder Dislocation Managed by Latarjet Procedure: A Prospective Study

**DOI:** 10.7759/cureus.21769

**Published:** 2022-01-31

**Authors:** Abhishek K Rai, Ajinkya R Bandebuche, Dixit Bansal, Devanshu Gupta, Ajay Naidu

**Affiliations:** 1 Department of Orthopaedics, Seth Gordhandas Sunderdas Medical College (GSMC) and the King Edward Memorial (KEM) Hospital, Mumbai, IND; 2 Department of Orthopaedics, Lokmanya Tilak Municipal General Hospital (LTMGH) and Sion Hospital, Mumbai, IND; 3 Department of Orthopaedics, Government Medical College, Amritsar, IND

**Keywords:** coracoid osteotomy, rowe score, ucla score, vas scale, latarjet procedure, anterior shoulder instability, chronic shoulder dislocation

## Abstract

The shoulder joint is the most common joint to undergo dislocation, with the anterior subtype being the most common. The most accepted definition of chronic dislocation is a shoulder joint that has remained dislocated for a minimum of three weeks. Due to rare presentation, there is a lack of consensus among surgeons regarding the optimal management option of chronic shoulder dislocation. The goal of this prospective study was to assess the efficacy of open reduction with Latarjet procedure in the management of chronic unreduced shoulder dislocation. A total of seven patients were included in this study. Five patients were males and two were females. The study was conducted in a single tertiary care centre between July 2015 and May 2018. All patients were managed by open reduction with the Latarjet procedure. The capsulolabral structures were repaired in all the cases. The post-operative functional outcome was assessed by shoulder range, Rowe score, and the University of California, Los Angeles (UCLA) score at regular intervals for a period of one year. There was a significant improvement in terms of pain relief and functional status of the patients. The patients were satisfied as they could do their daily routine activities without pain at a one-year follow-up. Early post-operative rehabilitation and physiotherapy are key to improving the functional range.

## Introduction

The shoulder joint is the most common joint to undergo dislocation, with the anterior subtype being the most common [1]. Most of the time, the shoulder dislocation is reduced manually by the patient himself or the surgeon in the outpatient department [2]. There is considerable debate in the literature about which shoulder dislocations are acute or chronic depending on the interval between injury and reduction. The most accepted definition of chronic dislocation is a shoulder joint that has remained dislocated for a minimum of three weeks [3]. Chronic anterior shoulder dislocation is an entity known to be rare in orthopaedics. Most commonly, it occurs in the elderly population due to soft tissue weakness and degeneration around the affected shoulder joint. Though rare, alcoholism, epilepsy, and repetitive trauma predispose a young patient to undergo chronic dislocation of the shoulder. Though few studies stress the point that chronic anterior dislocation does not limit the functional status of the shoulder joint, it does affect the function [4]. The associated injuries with shoulder dislocation are Bankart lesion, Hill-Sachs lesion, glenoid bone loss, acromion fracture, and proximal humeral fractures. The association of neurovascular injury in chronic dislocations are rarely reported [5]. As per the literature, various treatment modalities available are closed reduction, open reduction with Kirschner wires, Bankart repair, Latarjet procedure, hemiarthroplasty, and reverse shoulder hemiarthroplasty [6]. Due to the rarity of these infrequent chronic dislocations, little is known about the initial management and routine treatment protocol. The controversy still exists regarding the optimal treatment and most beneficial intervention of chronic anterior dislocation [7-9]. The Latarjet procedure, originally described in 1954, is effective in the management of chronic anterior shoulder dislocations if the glenoid bone loss exceeds 25%. The Latarjet procedure involves the osteotomy of the coracoid and its transfer along with the attached conjoint tendon to the anteroinferior aspect of the glenoid. The coracoid bone block along with the conjoint tendon acts as a strut that prevents re-dislocation of the shoulder joint [10]. This study highlights the effectiveness and functional outcome of patients with chronic anterior shoulder dislocation managed by the Latarjet procedure. The post-operative functional outcome was assessed by shoulder range, Rowe score, and the University of California, Los Angeles (UCLA) score.

## Materials and methods

The prospective study on seven patients was conducted at the department of orthopaedics of a tertiary care hospital with prior approval for the same from the Departmental Review Board and Institutional Ethics Committee before the initiation of the study (Institutional Review Board, dated 10 June 2015; approval number: IEC/OA/2015/38). The study includes all unilateral cases of chronic anterior dislocation of the shoulder managed by open reduction by the Latarjet procedure. Exclusion criteria were as follows: (i) acute shoulder dislocations of less than three weeks duration; (ii) recurrent anterior dislocation of the shoulder; (iii) chronic anterior dislocation of the shoulder associated with neuropathic arthropathy; (iv) congenital anterior dislocation of the shoulder; (v) patients with medical comorbidities who were surgically unfit; and (vi) bilateral shoulder dislocation.

**Figure 1:**
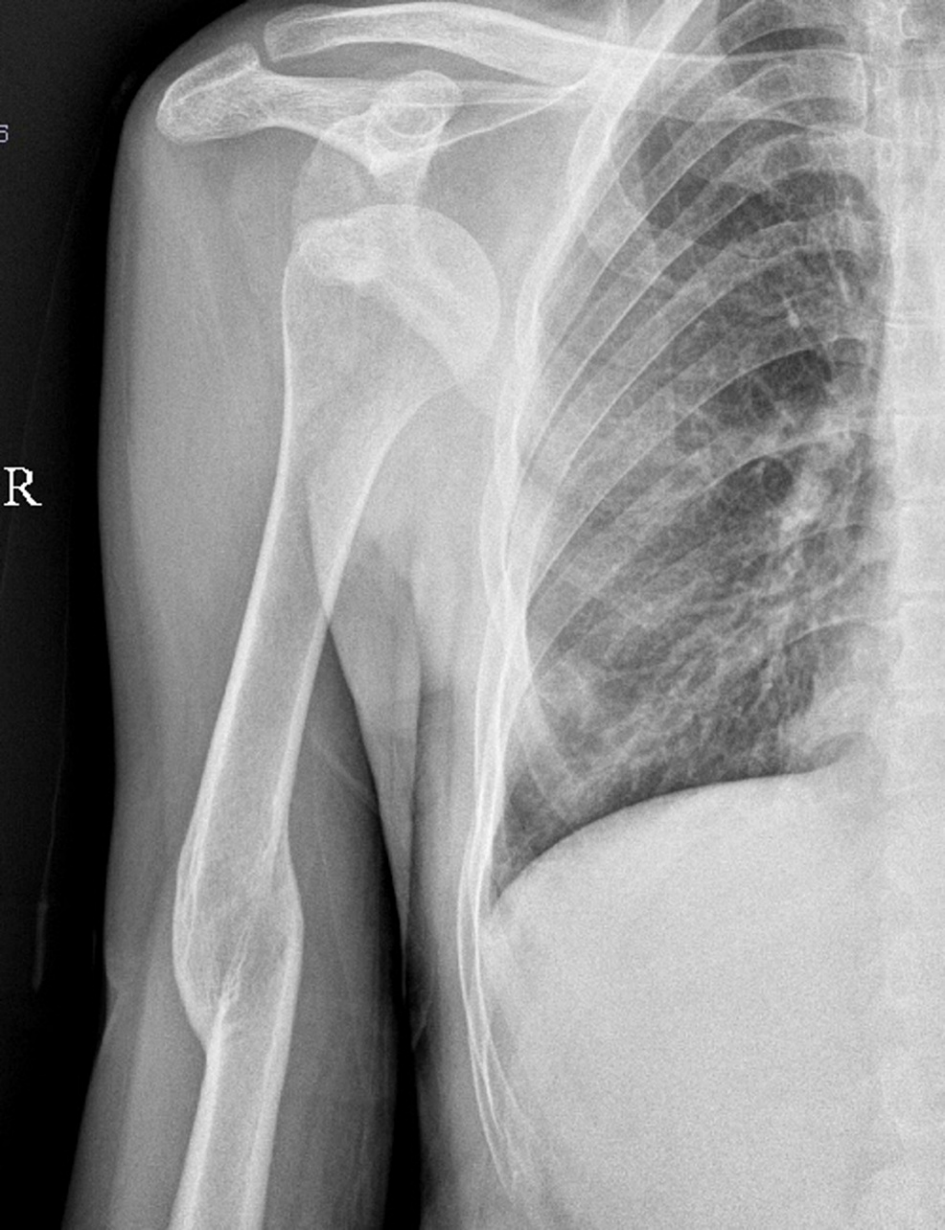
Pre-operative X-ray showing a subcoracoid subtype of anterior shoulder dislocation.

This was a single tertiary care centre, prospective study, which included all cases of chronic anterior dislocation of the shoulder between July 2015 and May 2018. Seven patients with chronic anterior shoulder dislocations (five males and two females) were included in the study (Table 1). The mean age was 47.1 years (range: 17-62 years). The duration of the dislocations ranged from 45 to 124 days (mean = 75.5 days). The mechanism of injury was road traffic accidents in three patients and fall while playing, fall from height, fall from stairs, and slip in the washroom in one patient each. The dislocation was diagnosed by clinical examination and confirmed on anteroposterior and axillary views (Figure 1). None of the patients had a distal neurovascular deficit. Computed tomography was not done in this study for financial constraints. All patients were right-hand dominant. We did not opt for a closed reduction in any of the patients. All patients were managed by open reduction and Latarjet procedure. The pain was quantified by the Visual Analog Scale (VAS) score. The post-operative functional outcome was assessed by shoulder range, Rowe score, and the University of California, Los Angeles (UCLA) score. The VAS score is a unidimensional pain measurement scale, which ranges from 0 to 10. The VAS score is evaluated as 0 (no pain), 1-3 (mild pain), 4-6 (moderate pain), and 7-10 (severe pain). The UCLA score evaluates the functional outcome of the shoulder joint based on combined objective and subjective parameters. The subscales of the UCLA score are pain, active forward flexion, the satisfaction of the patient, and function and strength of the forward flexion. The maximum score possible is 35, with values greater or equal to 27 are considered excellent and less than 27 are considered poor. Another score used in our study was the Rowe score for the functional status of the shoulder joint. The Rowe score can be interpreted as excellent (100-90), good (90-75), fair (75-51), and poor (50 or less). The patients were routinely followed up postoperatively at three weeks, six weeks, three months, six months, and 12 months for clinical evaluation and radiographs.

**Table 1:** Demographic details of all patients along with the mechanism of injury and the pre-operative duration in days.

Case No.	Age (years)	Gender	Mechanism of Injury	Involved shoulder	Pre-operative duration (days)
1	17	Female	Fall while playing on the ground	Right	45
2	42	Male	Road traffic accident	Right	70
3	60	Male	Fall from height	Right	60
4	52	Male	Road traffic accident	Left	124
5	48	Male	Fall from stairs	Left	90
6	62	Male	Slip in washroom	Right	84
7	49	Female	Road traffic accident	Right	56

Surgical technique

**Figure 2:**
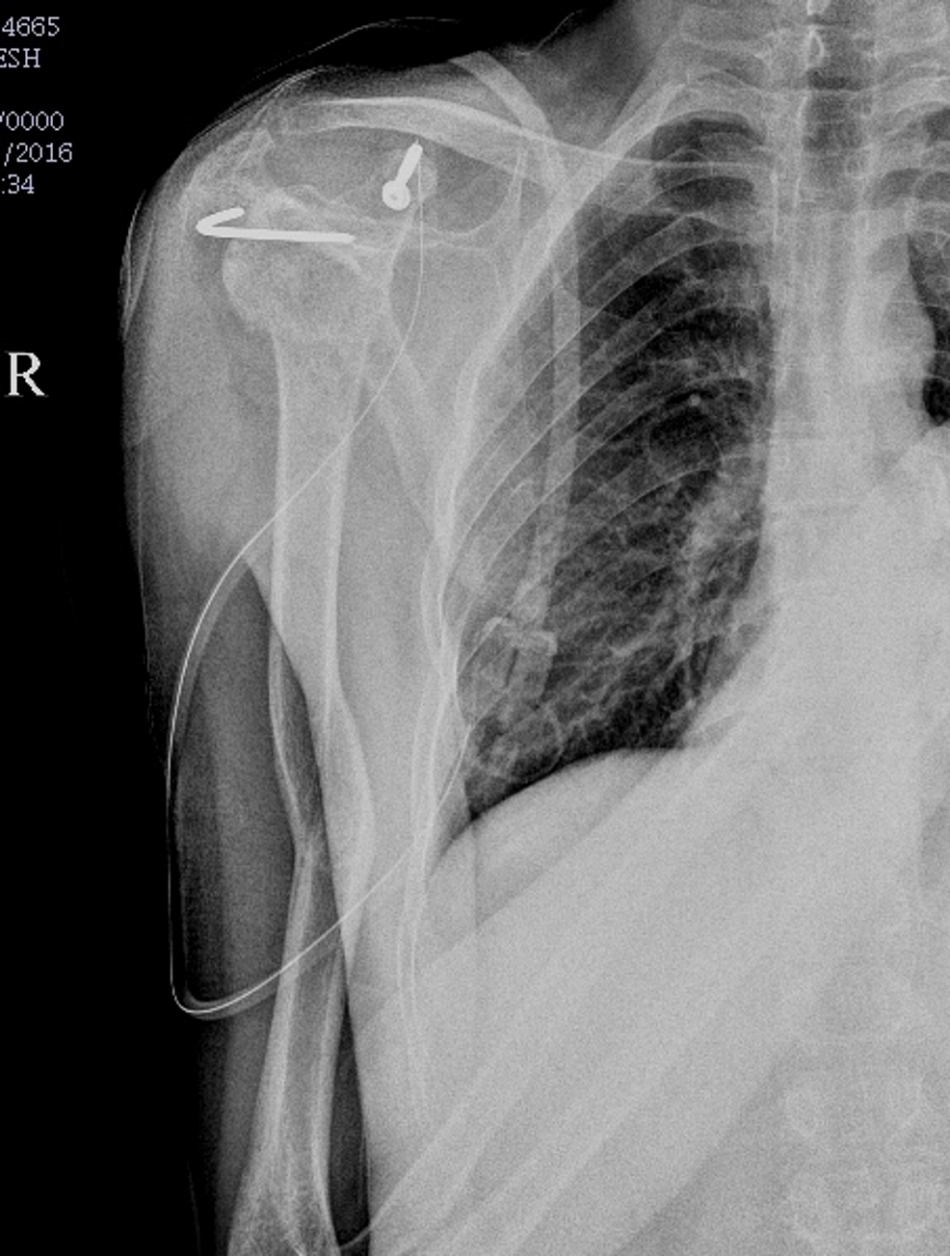
Immediate post-operative X-ray.

**Figure 3:**
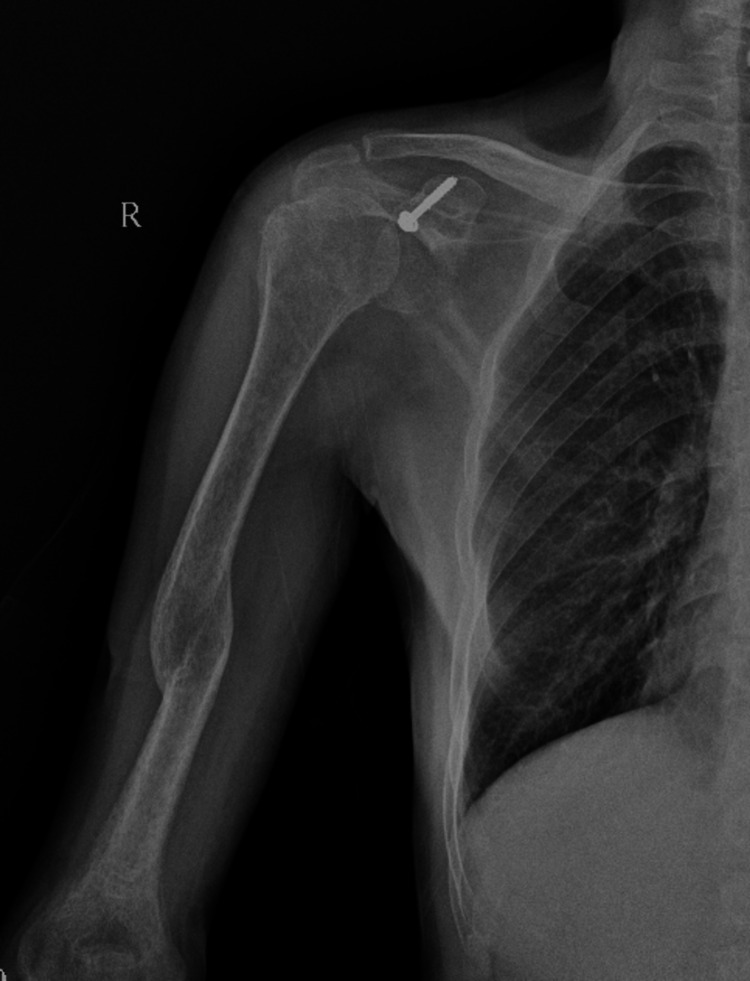
One-year follow-up X-ray showing well-reduced glenohumeral joint.

Under general anaesthesia, the patient was in a beach chair position. The deltopectoral approach was used. Coracoid osteotomy was performed at the base of the coracoid process after releasing the coracohumeral ligament laterally and pectoralis minor medially. The coracoid process along with the conjoint tendon retracted medially so as to expose underlined subscapularis muscle. The upper two-third part of the subscapularis muscle was cut after taking tag sutures to expose the humeral head. The glenoid cavity was approached by retracting the humeral head laterally with the Schanz pin and T-handle. Fibrosed tissue around the glenoid cavity was removed. The reduction was attempted with Kocher’s manoeuvre and levering the humeral head over Murphy’s skid. In all cases, the capsulolabral structure was repaired. The reduction was confirmed under intraoperative fluoroscopy. Subscapularis was approximated with sutures. Coracoid osteotomy was re-attached with a 4 mm CC screw with a washer (Figure 2). The patient's shoulder was placed in a splint for six weeks, post-operatively. The shoulder range of motion and rotator cuff strengthening exercises were started after six weeks. The radiographs (Figure 3), VAS score, Rowe score, and the UCLA score were interpreted at each follow-up.

## Results

**Figure 4:**
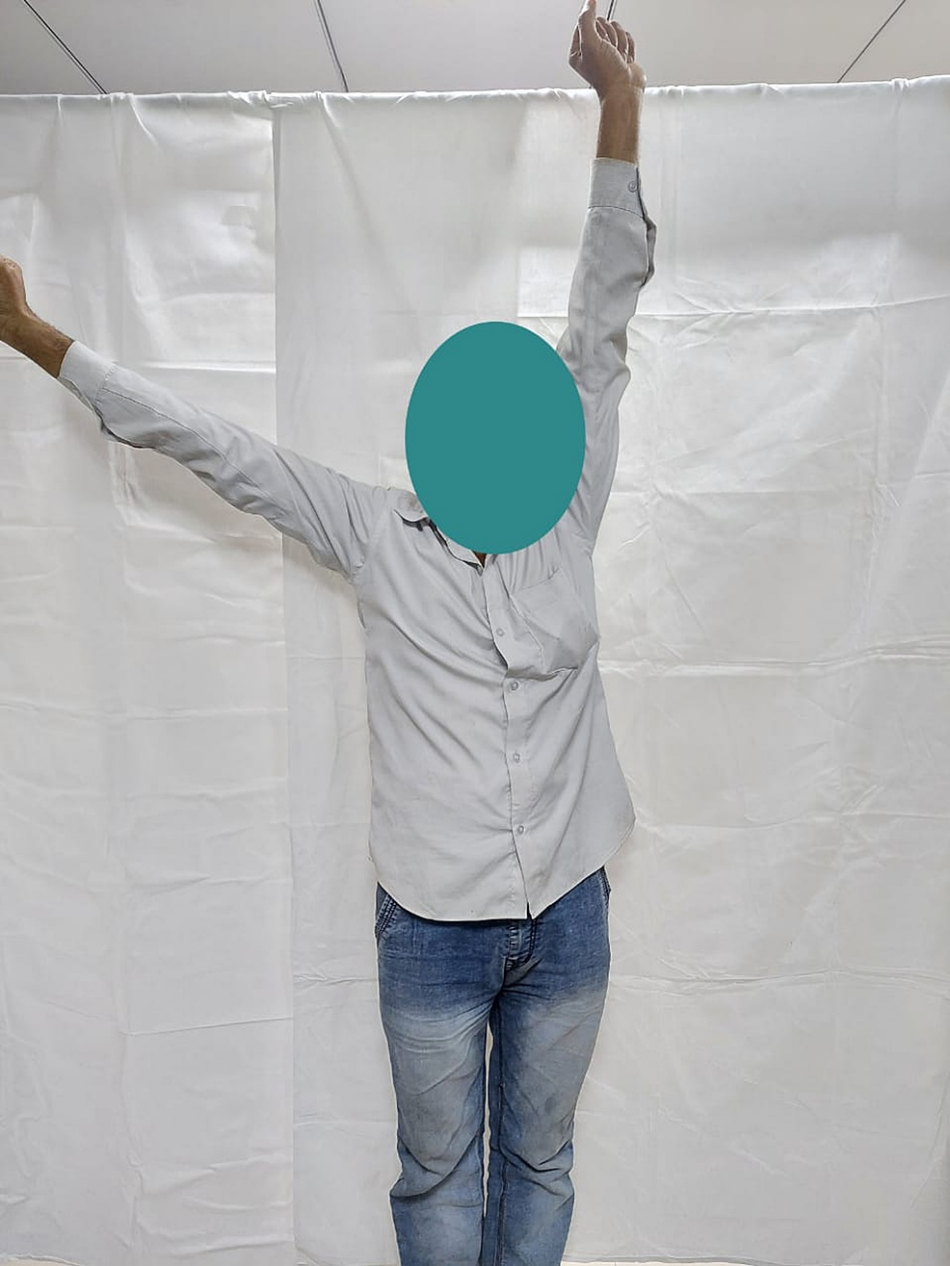
Abduction (110 degrees) of the right shoulder in a 42-year-old male at one-year follow-up.

**Figure 5:**
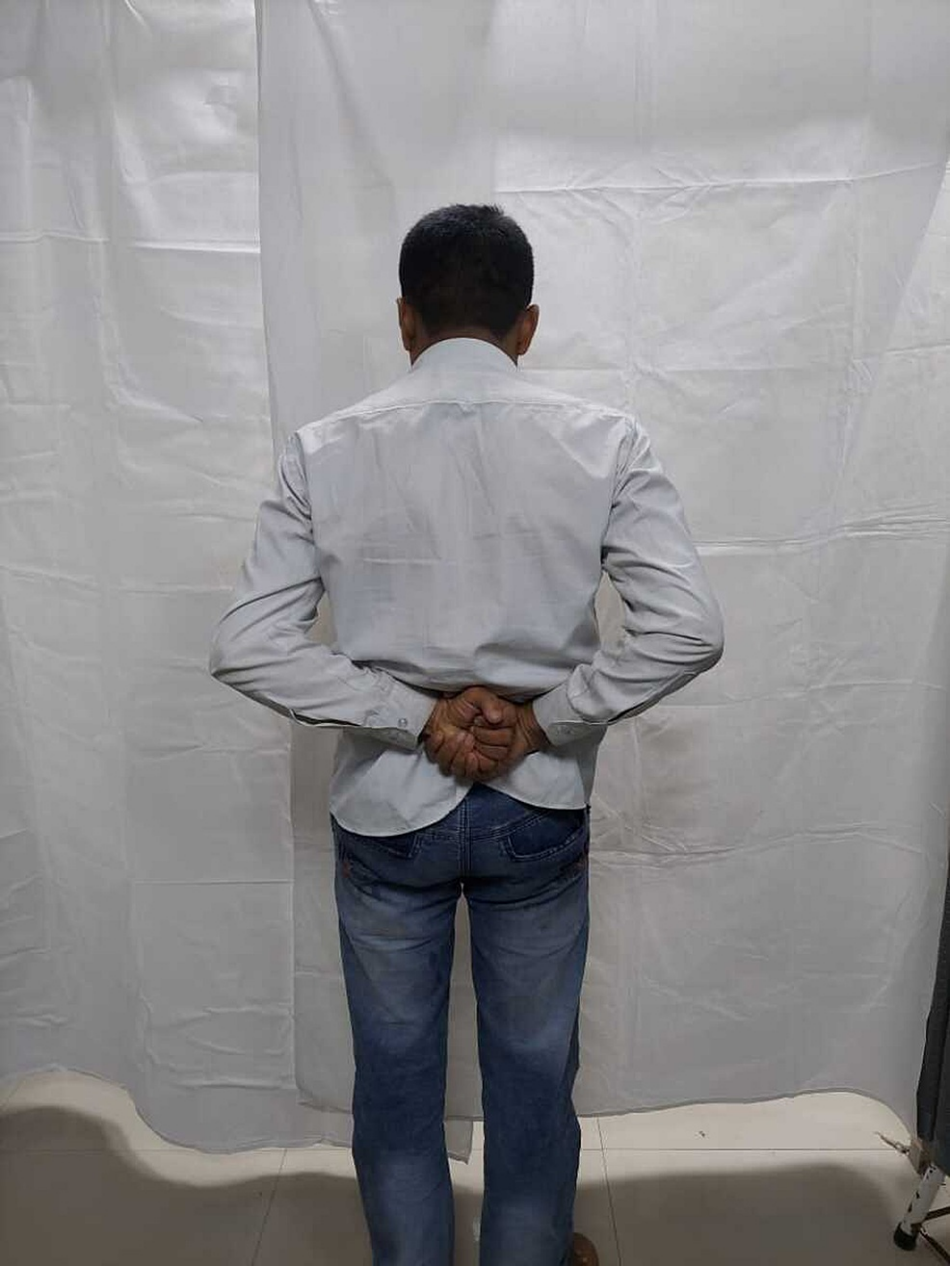
Internal rotation of the right shoulder in a 42-year-old male at one-year follow-up.

**Figure 6:**
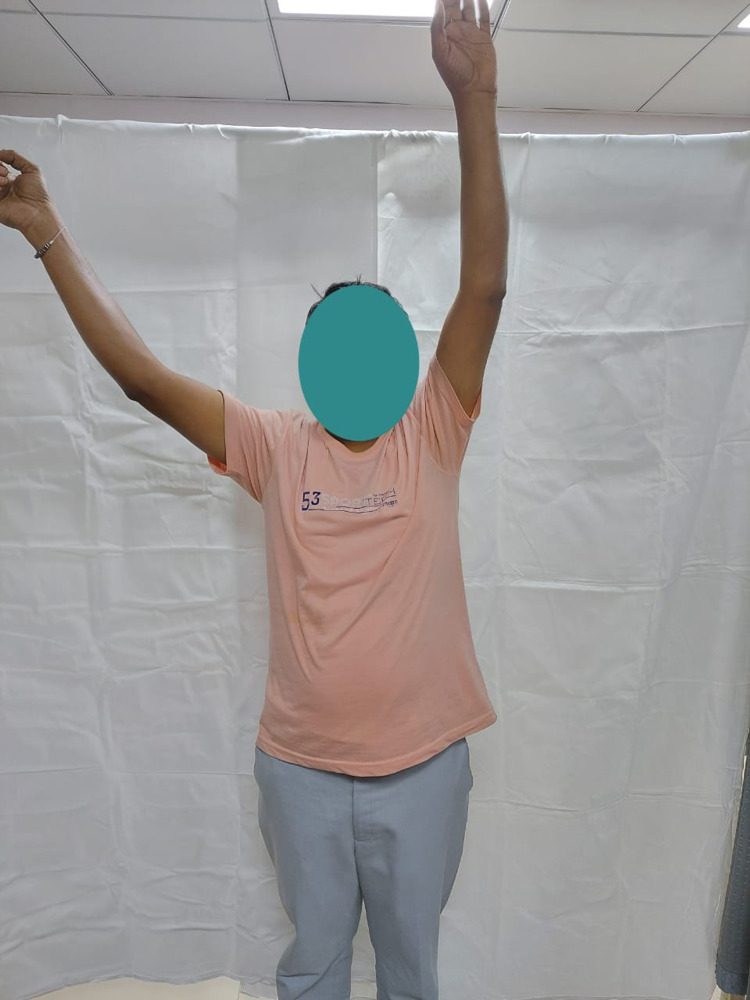
Abduction (130 degrees) of the right shoulder in a 60-year-old male at one-year follow-up.

**Figure 7:**
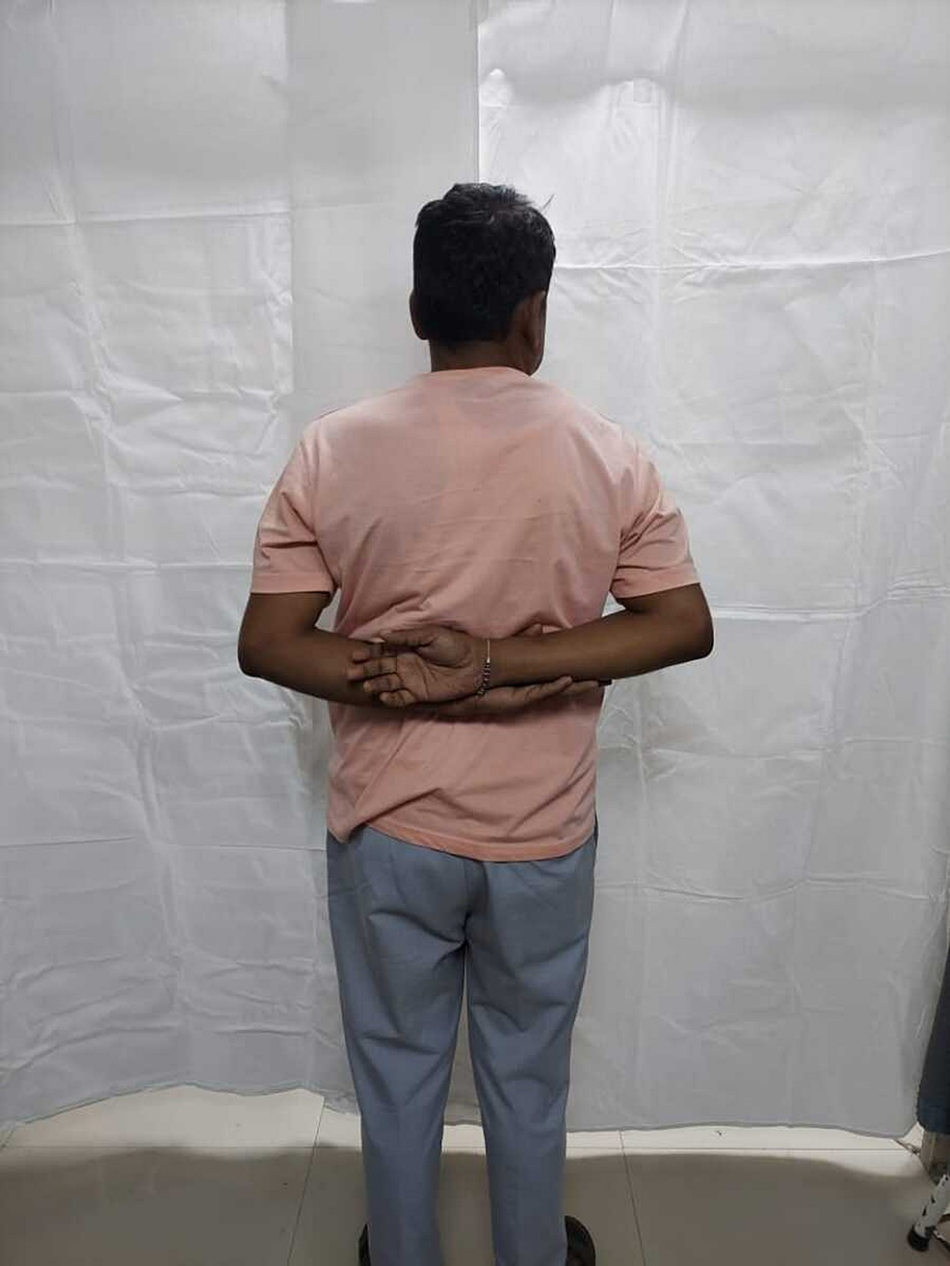
Internal rotation of the right shoulder in a 60-year-old male at one-year follow-up.

**Figure 8:**
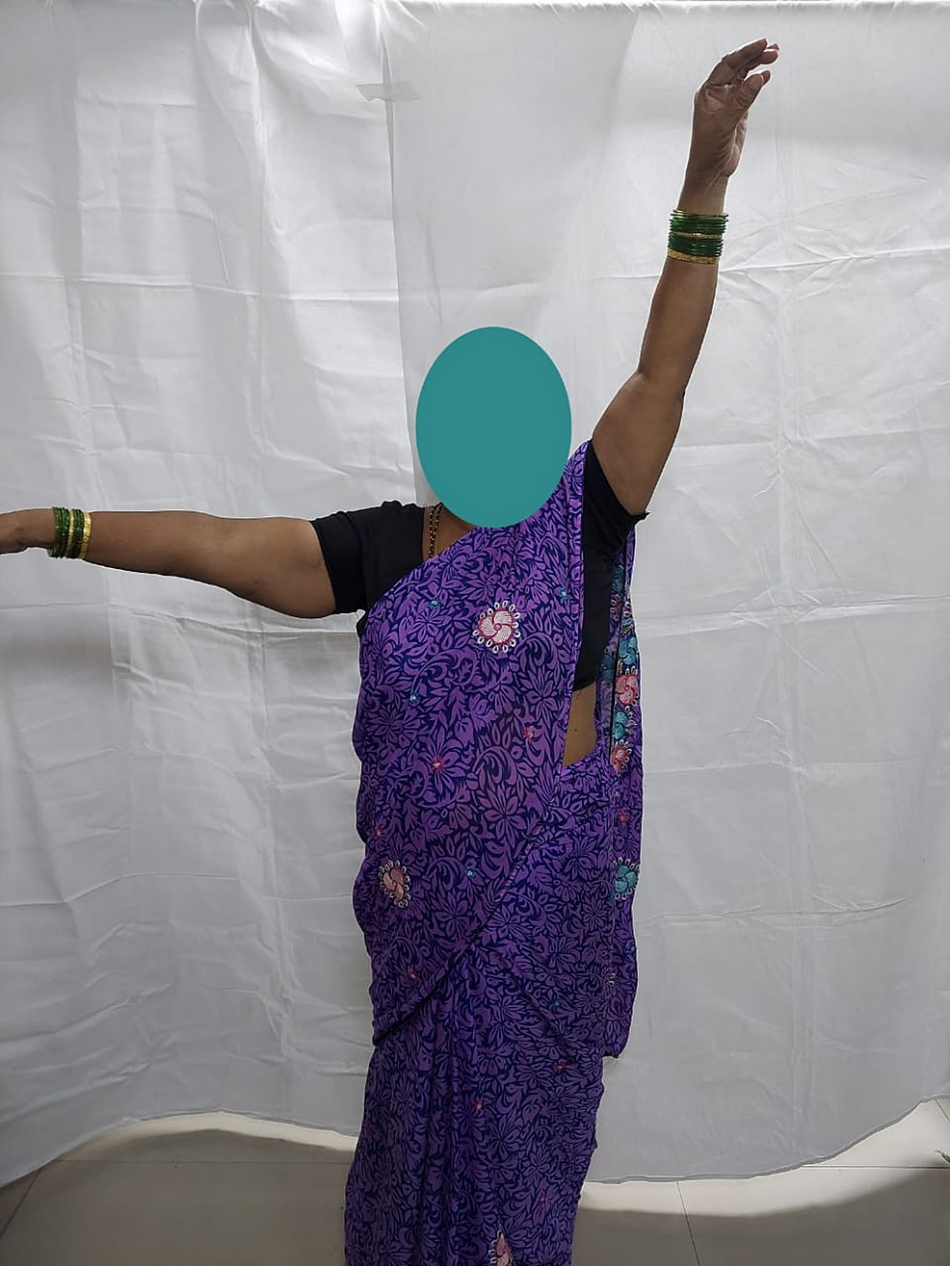
Abduction (90 degrees) of the right shoulder in a 49-year-old female at one-year follow-up.

**Figure 9:**
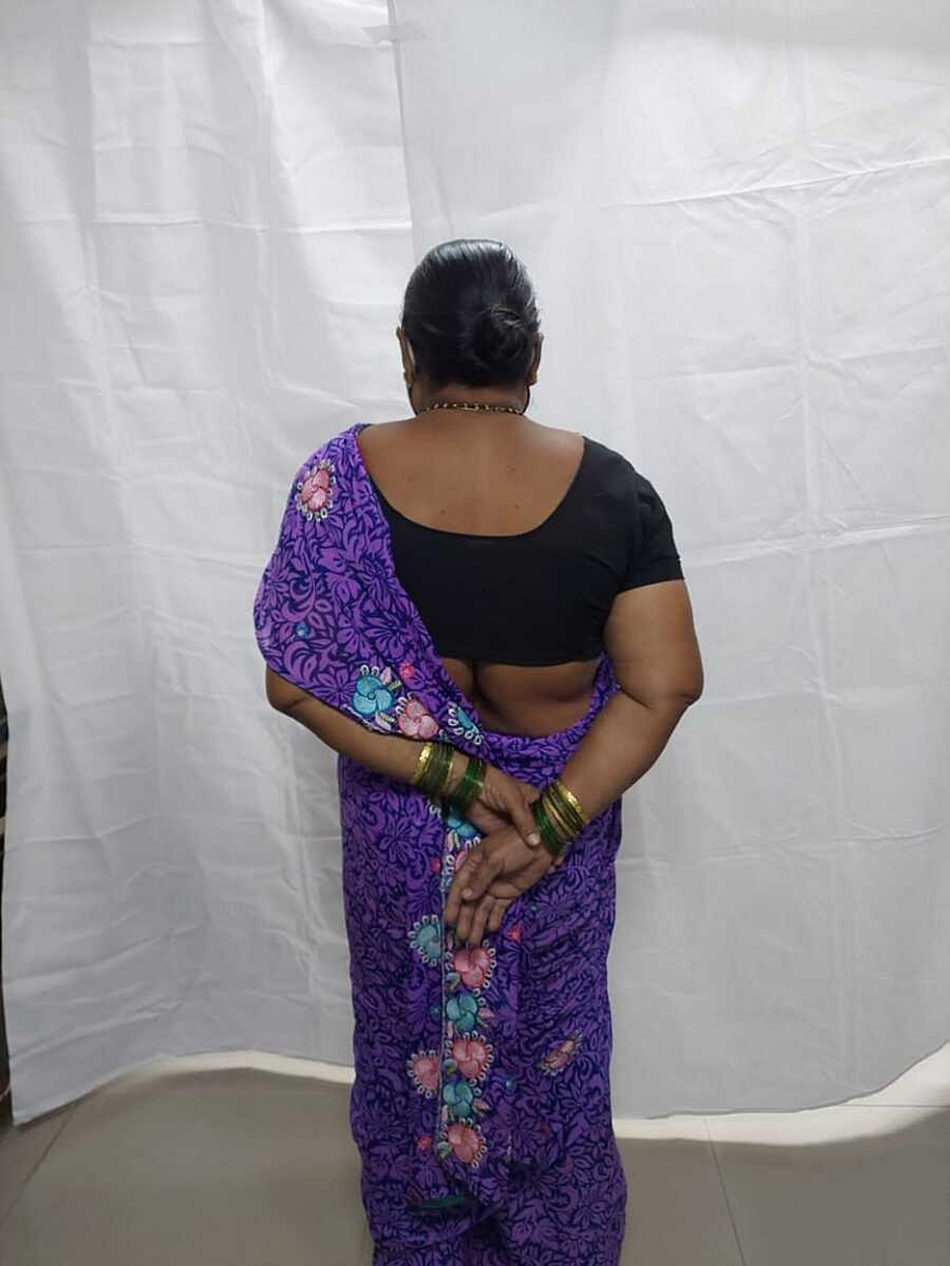
Internal rotation of the right shoulder in a 49-year-old female at one-year follow-up.

A total of seven patients who presented to our outpatient department during the study period were included in the study. There were five males (71%) and two females (29%). The mean age of the patients was 47.1 years (range: 17-62 years). The duration of the dislocations ranged from 45 to 124 days (mean = 75.5). The patients were routinely followed up postoperatively at three weeks, six weeks, three months, six months, and 12 months for clinical evaluation and radiographs (Table 2). The mean follow-up was 14.2 months (12-16 months). The VAS score pre-operatively was 6.5 ± 0.56, which reduced significantly to 2.2 ± 0.16 (p < 0.01) at 12 months follow up. The mean range of abduction pre-operatively was 37.7º ± 0.42º, which increased to 108.2º ± 2.48º (p < 0.01) at one-year follow-up. Similar trends were noticed in the UCLA and Rowe scores. The UCLA score increased from 8.4 ± 0.12 to 24.45 ± 1.22 (p < 0.01) at 12 months post-operatively. The Rowe score pre-operatively was 28.8 ± 0.34, and it increased significantly to 76.6 ± 2.44 (p < 0.01). At a follow-up period of more than a year, none of the patients had re-dislocations or superficial or deep infections. None of the cases had a procedure-related neurovascular injury. Two patients complained of numbness on the upper lateral aspect of the shoulder for whom we suspected an injury to the axillary nerve but both recovered at six weeks follow up. All patients were satisfied with the surgery and the functional outcome they achieved in a one-year follow-up. The range of shoulder movement was measured for abduction and internal rotation at one-year follow-up (Figures 4-9).

**Table 2:** Detailed post-operative pain outcome in terms of VAS score and functional status in terms of shoulder abduction, UCLA score, and Rowe score. * Values are significant; p < 0.01. VAS, Visual Analog Scale; UCLA, University of California, Los Angeles.

Evaluation items	Pre-operative	Post-operative 3 weeks	Post-operative 6 weeks	Post-operative 3 months	Post-operative 6 months	Post-operative 12 months
VAS	6.5 ± 0.56	5.8 ± 0.42	4.2 ± 0.52*	3.6 ± 0.22*	2.8 ± 0.18*	2.2 ± 0.16*
Abduction (degrees)	37.7 ± 0.42	42.6 ± 0.34	54.8 ± 1.22	70.6 +- 1.71*	90.2 ± 2.42*	108.2 ± 2.48*
UCLA	8.4 ± 0.12	9.8 ± 0.18	11.6 ± 0.24	15.8 ± 1.18*	18.6 ± 1.42*	24.45 ± 1.22*
Rowe score	28.8 ± 0.34	34.6 ± 0.62	44.2 ± 1.52*	56.6 ± 2.24*	64.4 ± 1.34*	76.6 ± 2.44*

## Discussion

The shoulder joint is the most vulnerable and thus the most common joint to undergo dislocation. Shoulder dislocation can occur in any direction, anterior being the most common. Various authors have defined chronic dislocations in different ways. Few authors suggested that any dislocation that has not been reduced in a period of 24 hours should be considered chronic dislocation [11]. Goga defined chronic dislocation as one which is one or more weeks old in an unreduced position [12]. The most widely accepted definition of chronic shoulder dislocation is a joint that has remained unreduced for a period of three or more weeks [13]. Chronic shoulder dislocation is a rare entity. Most of the studies have shown its prevalence ranging from 0.10% to 0.18%. Most commonly, it occurs in the elderly population due to soft tissue weakness and degeneration of rotator cuff muscles around the affected shoulder joint. Soft tissue imbalance due to thinning and lengthening of musculotendinous structure around shoulder joint poses risk for re-dislocation. Though rare, alcoholism, epilepsy, and repetitive trauma predispose a young patient to undergo chronic dislocation of the shoulder. The mechanism of injury was road traffic accidents in three patients and fall while playing, fall from height, fall from stairs, and slip in the washroom in one patient each. None of the patients had a history of previous dislocations or generalized ligamentous laxity. All cases reported in our study were chronic anterior dislocation with subcoracoid subtype. Most of the cases were neglected by the patient and were managed conservatively by some quacks in the form of massages, bone setting, or homemade slings. The major complaint of the patients was severe pain and limitation of functional activities. The choice of treatment depends upon various factors, which include duration of dislocation, associated musculoskeletal injuries, the status of the rotator cuff, hand dominance, previous operative and nonoperative management, and the functional demands of the patient. The high failure rates after conservative management are due to soft tissue interposition and associated bony block. In the past, patients with chronic dislocations were managed conservatively provided they had a functional range of motion. But few recent studies have shown that open reduction always has a good functional outcome compared to conservative management. As per the literature, various treatment modalities available are closed reduction, open reduction with Kirschner wires, Bankart repair, Latarjet procedure, hemiarthroplasty, and reverse shoulder hemiarthroplasty.

Babalola et al. managed nine cases of chronic anterior shoulder dislocation by open reduction and fixation with Kirschner wires in five patients and non-operative management in four patients [14]. They concluded that operative intervention combined with meticulous physiotherapy contributes to a favourable outcome. Yang et al. did a retrospective study on 20 shoulders of 18 patients, of whom 16 were managed by Latarjet procedure and four patients by Bristow procedure [15]. They found that there was a significant improvement in range of motion, VAS score, UCLA score, and American Shoulder and Elbow Surgeons (ASES) score in all the patients. They concluded that coracoid osteotomy yields significant improvement in the functional outcome. Lubis et al. presented a case of a 10-month-old anterior shoulder dislocation managed by open reduction with Latarjet procedure [16]. They concluded that coracoid osteotomy helps in preventing re-dislocation and improves the functional status of the shoulder joint. Li and Jiang stated that hemiarthroplasty is a better option in cases of chronic shoulder dislocation with or without coracoid osteotomy [17]. Kohan et al. advocated that reverse total shoulder arthroplasty prevents the incidence of re-dislocation and improves the range of shoulder in cases of chronic shoulder dislocation [18]. Neurovascular injury can occur in Latarjet procedure, commonly during coracoid osteotomy. The musculoskeletal nerve and axillary nerve are prone to be injured.

In our study, we managed all patients by open reduction with the Latarjet procedure. The capsulolabral structures were repaired in all cases. Two patients complained of numbness over the lateral aspect of the upper arm, which gradually resolved by the six weeks follow up. The outcome in terms of VAS score, UCLA score, Rowe score, and the functional status of the shoulder joint was significantly better during the 12-month post-operative period. All patients were satisfied with the outcome in terms of pain relief and functional improvement of the shoulder joint.

There are a few limitations of our study. First, there was no control group in our study for comparison. Second, considering the rarity of this condition, the sample size was small to comment on the efficacy of the Latarjet procedure in the management of chronic shoulder dislocation. Third, the follow-up period of this study was short, i.e., around 14 months. Fourth, a CT scan needs to be done to quantify bone loss.

## Conclusions

Open reduction with Latarjet procedure is a highly successful procedure in the management of chronic anterior shoulder dislocation. The procedure decreases pain and improves the functional status of the patient. The associated musculoskeletal injury needs to be addressed properly for improving the outcome. Due to rare presentation, there is a lack of consensus among surgeons regarding the optimal management option of chronic shoulder dislocation. There is a need for a study with higher sample size and long-term follow up. Early post-operative rehabilitation and physiotherapy are key to improving the functional range. In conclusion, although it is under debate regarding the optimal management option of an old unreduced shoulder dislocation, our study advocates that open reduction with Latarjet procedure offers a decrease in pain and satisfies the patient with the improvement in joint movement, which enables them to perform their daily activities.
